# H_2_S-Mediated Protein *S*-Sulfhydration: A Prediction for Its Formation and Regulation

**DOI:** 10.3390/molecules22081334

**Published:** 2017-08-11

**Authors:** Youngjun Ju, Ming Fu, Eric Stokes, Lingyun Wu, Guangdong Yang

**Affiliations:** 1 School of Kinesiology, Lakehead University, Thunder Bay, ON P7B 5E1, Canada; yju@lakeheadu.ca; 2 Cardiovascular and Metabolic Research Unit, Laurentian University, 935 Ramsey Lake Rd, Sudbury, ON P3E 2C6, Canada; mfu@laurentian.ca (M.F.); estokes@laurentian.ca (E.S.); lwu2@laurentian.ca (L.W.); 3 School of Human Kinetics, Laurentian University, 935 Ramsey Lake Rd, Sudbury, ON P3E 2C6, Canada; 4 Health Science North Research Institute, 41 Ramsey Lake Rd, Sudbury, ON P3E 5J1, Canada; 5 The Department of Biology, Laurentian University, 935 Ramsey Lake Rd, Sudbury, ON P3E 2C6, Canada; 6 The Department of Chemistry and Biochemistry, Laurentian University, 935 Ramsey Lake Rd, Sudbury, ON P3E 2C6, Canada

**Keywords:** hydrogen sulfide, nitric oxide, cysteine, *S*-sulfhydration, *S*-nitrosylation

## Abstract

Protein *S*-sulfhydration is a newly discovered post-translational modification of specific cysteine residue(s) in target proteins, which is involved in a broad range of cellular functions and metabolic pathways. By changing local conformation and the final activity of target proteins, *S*-sulfhydration is believed to mediate most cellular responses initiated by H_2_S, a novel gasotransmitter. In comparison to protein *S*-sulfhydration, nitric oxide-mediated protein *S*-nitrosylation has been extensively investigated, including its formation, regulation, transfer and metabolism. Although the investigation on the regulatory mechanisms associated with protein *S*-sulfhydration is still in its infancy, accumulated evidence suggested that protein *S*-sulfhydration may share similar chemical features with protein *S*-nitrosylation. Glutathione persulfide acts as a major donor for protein *S*-sulfhydration. Here, we review the present knowledge on protein *S*-sulfhydration, and also predict its formation and regulation mechanisms based on the knowledge from protein *S*-nitrosylation.

## 1. Introduction

Over the past decades, the modification of cysteine residues by *S*-nitrosylation has been extensively studied. Similar to phosphorylation, *S*-nitrosylation is a ubiquitous post-translational modification by selective addition of nitric oxide (NO) moiety to specific cysteine residue(s) in target proteins forming *S*-nitrosothiol (SNO). So far, many techniques have been developed to identify the nitrosylated cysteine residues. A modified biotin switch assay (BSA) was first utilized to detect *S*-nitrosylated cysteine through the replacement of labile NO on protein cysteine residues with a stable biotin moiety [1]. In addition, the application of other methods such as tandem mass spectrometry (MS/MS) [1,2] and SNOSID (SNO Site Identification) [3] have been developed to detect and analyze the cysteine residues of *S*-nitrosylation on proteins. With these experimental approaches, many studies have been trying to explore the chemical features and regulatory mechanisms of cysteine *S*-nitrosylation [4,5,6,7,8]. The acid–base motif, in particular, offers a potentially promising concept for the specificity of cysteine involved in *S*-nitrosylation formation [7,8,9]. It also has been demonstrated that *S*-nitrosylated cysteine may often be flanked by hydrophobic amino acids, showing high surface exposure, high reactivity, and low pKa [8,9].

*S*-sulfhydration, or *S*-persulfidation, is a newly discovered protein post-translational modification by yielding a hydropersulfide moiety (–SSH) or polysulfide in the active cysteine residues, which mediates most of the cellular functions induced by hydrogen sulfide (H_2_S), a novel member in the gasotransmitter family together with NO and carbon monoxide [10,11,12,13,14]. Since the first finding of *S*-sulfhydration on proteins was described in 2009 [10], many proteins have been reported to be *S*-sulfhydrated and involved in the physiological and pathological functions of H_2_S. H_2_S acts as an endothelium-deriving relaxing factor (EDRF) through *S*-sulfhydration of potassium channel proteins [15]. *S*-sulfhydration of Keap1 provides protection against cellular senescence via the regulation of Nrf2 activity [16]. Recently, it was found that *S*-sulfhydration of MEK1 is associated with repairing damaged DNA inside the cell [17]. eNOS *S*-sulfhydration regulates eNOS activity through the regulation of eNOS dimerization [18]. In addition, abnormal protein *S*-sulfhydration has been found to be involved in multiple sclerosis [19], antioxidants [20], neuroprotection [21], and endoplasmic reticulum stress response [22] by altering enzymatic activity, protein localization, protein-protein interactions, and protein stability, etc. Aside from protein modification by *S*-sulfhydration, H_2_S redox interaction of heme proteins is another important pathway in sulfide biochemistry [23], which will not be discussed in this review.

In comparison to protein *S*-sulfhydration, protein *S*-nitrosylation has been extensively studied, including its formation, regulation, transfer and metabolism [9,24]. The investigation of the regulatory mechanisms associated with protein *S*-sulfhydration is still in its infancy, little is known about the chemical features and biochemical stability of protein *S*-sulfhydration. Protein *S*-sulfhydration and *S*-nitrosylation share many similarities in terms of their chemical and biological features [11,12,13,25]. In this review, we summarize the present knowledge on protein *S*-sulfhydration, and also predict its formation and regulation mechanism based on the concepts of protein *S*-nitrosylation. 

## 2. Potential Forming Mechanisms of Protein *S*-Sulfhydration

### 2.1. Protein S-Sulfhydration Detection

It was predicted that one-third of proteins could be modified forming *S*-sulfhydration, suggesting that *S*-sulfhydration is a highly prevalent protein post-translational modification [10,11]. *S*-sulfhydration usually increases the reactivity of target proteins, whereas *S*-nitrosylation often decreases protein activity [14,25,26]. In terms of its instability and higher nucleophilic characteristic, the detection of cysteine *S*-sulfhydration is quite challenging. Until now, several methods have been developed to detect *S*-sulfhydration. Based on the detection method from *S*-nitrosylation, a modified biotin switch assay was first utilized for detection of *S*-sulfhydration [10]. In this method, methyl methanethiosulfonate (MMTS) was used to block unmodified cysteine residues, then persulfide group(s) were labeled with biotin. Through purification of biotinylated proteins with streptavidin conjugates and Western blotting with a specific antibody, protein *S*-sulfhydration can be determined. Later studies found that MMTS can also interact with the persulfide group, which would lead to a reduced signal of protein *S*-sulfhydration [27,28]. A second method was then established by using fluorescent thiol modifying reagent Cy5-conjugated maleimide to selectively label both modified persulfide and the unmodified free thiol group [29]. Dithiothreitol (DTT) incubation reduces only persulfide but not unmodified cysteines, resulting in a lower intensity of fluorescent signal, which can be quantified to analyze the level of protein *S*-sulfhydration. A weakness of this method is that maleimide assay would not distinguish *S*-sulfhydration from other ways of cysteine modification, such as *S*-nitrosylation and *S*-glutathionylation [12]. Different with biotin switch assay, a new tag switch assay uses methylsulfonylbenzothiazole (MSBT), an aromatic thiol-blocking reagent, to block both free thiols and persulfides [27]. Afterwards, a nucleophilic tag-switch reagent (cyanoacetic acid nucleophile) was added for only labeling the persulfide groups, which are then enriched using streptavidin conjugates and analyzed by Western blot. A recent improved tag switch assay was reported to select two new cyanoacetic acid derivatives with the fluorescent moiety to increase the sensitivity of detection [28]. Although the selectivity of protein *S*-sulfhydration detection is higher, with a tag switch assay it is difficult to detect the cross-reactivity of persulfide with other cysteine post-translational modification [28,29]. To detect polysulfide in target protein, two novel and highly specific methods were established, named as polyethylene glycol-conjugated maleimide-labeling gel shift assay (PMSA) and protein persulfide detection protocol (ProPerDp) [30,31], respectively. 

### 2.2. Acid–Base Motif in Protein S-Sulfhydration

**Figure 1:**
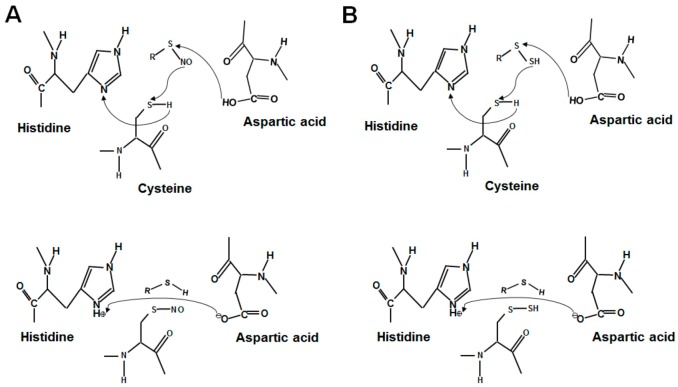
The proposed forming mechanisms of protein *S*-nitrosylation and *S*-sulfhydration through the acid–base motif. (**A**) The proposed mechanism of *S*-nitrosylation in the acid–base motif. (**B**) The proposed mechanism for *S*-sulfhydration. The regulatory mechanism for *S*-nitrosylation and *S*-sulfhydration is assisted by neighboring acid (histidine) and base (aspartic acid) amino acids.

Cysteine plays a number of important roles in regulating cellular functions through its thiol functional group. The number and location of cysteine residues in different proteins is varying. It is a big challenge to determine the specificity of cysteine in target protein for *S*-sulfhydration [29]. Based on the analysis of NO transfer in many proteins, the acid-base motif was suggested as a site for forming protein *S*-nitrosylation [7,8,9,32]. Electrostatic interactions of particular cysteine with nearby acid-base amino acids may alter thiol reactivity and confer structural instability. In the acid-base motif, donor molecules are facilitated to form protein *S*-nitrosylation (Figure 1A). The proposed donor molecules for protein *S*-nitrosylation are nitrosothiols (RSNO). Nitrosoglutathione (GSNO) is a well-known endogenous RSNO catalyzed by GSNO reductase (GSNOR), and GSNOR/GSNO system is critically involved in NO signaling by maintaining a pool of NO inside the cell [33,34]. Aside from GSNO, nitrosocysteine (CSNO) has also been shown to act as a bioavailable source of NO and to contribute to protein *S*-nitrosylation [35,36,37]. In addition, Ascenzi et al. proposed that formation of protein *S*-nitrosylation is dependent on the 3D structure of target *S*-nitrosylated cysteine residues but not on the linear sequence of amino acids in the protein (Figure 1A) [7]. 

As of today, the mechanism by which H_2_S targets specific protein thiols for *S*-sulfhydration remains unknown in comparison to protein *S*-nitrosylation. Direct reaction of H_2_S and free thiol is impossible in consideration of the thermodynamic constrains [25]. Polysulfides recently emerged as potential mediators of H_2_S signaling [38]. A direct correlation between glutathione persulfide (GSSH) and protein *S*-sulfhydration has been suggested [28,39,40]. Similar to GSNOR in the regulation of GSNO in the cells, a mitochondrial persulfide dioxygenase enzyme, ETHE1, mediates the generation of GSSH [39,41]. Ida et al. recently confirmed that that GSSH could be an intermediate of the mitochondrial H_2_S oxidation pathway [40]. The cysteine residues with low pKa exist as thiolate anions under normal conditions, are more easily attacked by various oxidants and are susceptible to *S*-sulfhydration [25]. Therefore, the acid-base motif might provide a potential explanation for the forming mechanism of protein *S*-sulfhydration, with GSSH acting as a donor of H_2_S for protein *S*-sulfhydration (Figure 1B). Furthermore, the highly efficient formation for protein *S*-sulfhydration mediated by H_2_S in vitro has been shown to occur through the addition of sulfane-sulfur from a small molecule of polysulfide, such as GSSH, rather than from SH^−^ as the primary thiol adduct [42]. Sulfane-sulfur is a good target for nucleophilic attack [43]. It is predicted that GSSH or CysSSH have higher nucleophilicity than parental GSH or cysteine. These reactive species improve oxidative stress by scavenging reactive oxygen species (ROS) and electrophiles, etc. [44]. Protein *S*-sulfhydration or polysulfidation somehow protect protein thiol residues from oxidants and electrophiles-induced damage [44]. It could be further implied that persulfide molecules may be involved in the regulatory mechanism of protein *S*-sulfhydration through the acid–base motif within spatial proximity to thiol groups [7,32]. The mediation of ATP level, pH value, oxygen level, and surrounding ionic strength may also be involved in the formation and regulation of cysteine *S*-sulfhydration, which needs to be tested further [26,43]. In addition, with H_2_S it may be difficult to directly reduce the disulfide-bond inside protein forming *S*-sulfhydration, since the reaction of disulfides with sulfide is a highly system-specific process from both thermodynamic and kinetic aspects [45].

### 2.3. Transsulfhydration via Protein–Protein Interaction

Thioredoxin (Trx) is one of the main disulfide reductase systems inside the cells together with thioredoxin reductase and NADPH, and has a wide variety of biological actions [46]. The regulation of protein *S*-nitrosylation by Trx1 has been reported [47,48,49,50]. It was suggested that Trx1 acts as a denitrosylase and/or transnitrosylase depending on the redox status of different cysteine residues in Trx1 (Table 1). Trx1 mediates denitrosylation of caspase-3, and the denitrosylation activity of Trx1 is dependent on the cysteine 32 and 35 in Trx1 [51]. *S*-nitrosylation of NF-κB inhibits its activity and Trx1 increases cytokine-induced NF-κB activation through the denitrosylation [46]. Differently, the transnitrosylation activity of Trx1 relies on cysteine 69 and 73 in Trx1. Trx1 itself is basically *S*-nitrosylated and *S*-nitrosylation of Trx1 transnitrosylates proteins, such as caspase-3 and apoptosis signal-regulating kinase 1 (ASK1) [52,53]. In this case, cysteine 73 of Trx1 plays an important role for the transnitrosylation of target proteins via direct interaction with Trx1 [51]. Along with Trx1, glyceraldehyde 3-phosphate dehydrogenase (GAPDH) also possesses the activity of transnitrosylation. Many nuclear proteins have been shown to be targeted by GAPDH for transnitrosylation, including the deacetylating enzyme sirtuin-1 (SIRT1), histone deacetylase-2 (HDAC2), and DNA-activated protein kinase (DNA-PK), etc. [54]. Furthermore, transnitrosylation of haemoglobin to the anion exchanger AE1 in the plasma membrane of red blood cells has also been reported [55].

**Figure 2:**
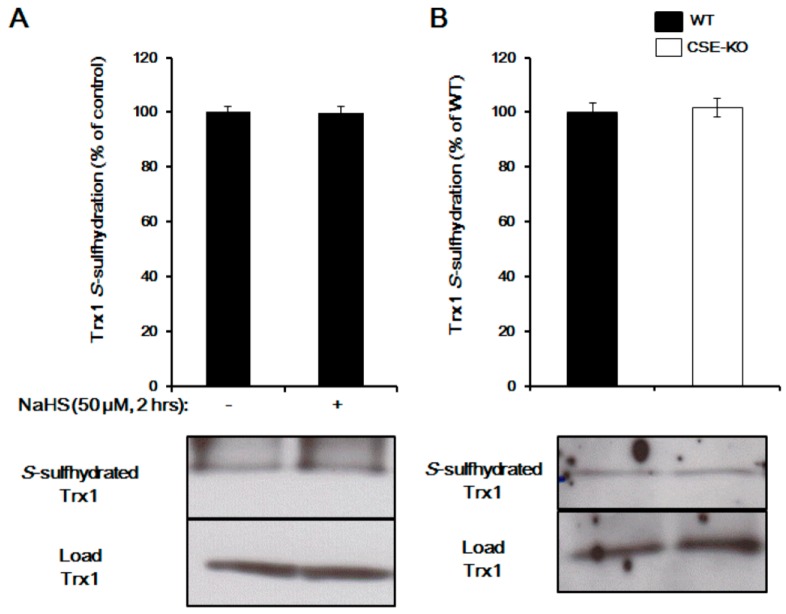
Trx1 is not a target for *S*-sulfhydration. (**A**) Trx1 *S*-sulfhdyration was measured in NaHS-treated HepG2 cells (50 µM for 2 h) by biotin switch assay. *n* = 4. (**B**) Trx1 *S*-sulfhydration was determined in liver tissues from both wild-type and CSE knockout mice. *n* = 4. CSE, cystathionine gamma-lyase; KO, knockout; Trx1, thioredoxin 1; WT, wild-type.

Trx1 regulation of protein *S*-sulfhydration has also been reported [28,46,57]. By interacting with the two redox active cysteine residues at its active site, Trx1 has the ability to bind with 3-mercaptopyruvate sulfurtransferase (3MST) to generate H_2_S, pointing to the possibility of Trx1 to break down the double-sulfide bond [46]. A later study proved that Trx1 reduced H_2_S-stimulated PTP1B *S*-sulfhydration [22]. Recombinant Trx1 showed a very high reactivity in cleaving protein persulfides and releasing H_2_S, while inhibition of the Trx1 system caused an increase in intracellular persulfides [28]. We recently provided evidence that Trx1 desulfhydrates pyruvate carboxylase and GAPDH, suggesting that Trx1 indeed acts as a *S*-desulfhydrase and controls H_2_S signaling [57]. Trx1 attenuates cysteine *S*-sulfhydration by direct interaction with *S*-sulfhydrated proteins at the Trp–Cys^32^–Gly–Pro–Cys^35^ motif. Deficiency of TrxR1 in mouse livers markedly elevated persulfide level, further indicating the distinct roles of the Trx systems in regulating protein *S*-sulfhydration or persulfide [31]. So far, there is no report about protein transsulfhydration activity of Trx1. We found that Trx1 is basically *S*-sulfhydrated, while Trx1 *S*-sulfhydration is not altered by exogenously applied NaHS or knockout of cystathionine gamma-lyase (CSE, a H_2_S-generating gene) (Figure 2), suggesting that Trx1 is not involved in protein transsulfhydration. Future studies need to be performed to find the proteins/enzymes/factors involved in protein transsulfhydration, which would help to understand the biological effect of H_2_S in both health and disease.

### 2.4. Protein S-Sulfhydration from Oxidized Cysteine (S–OH)

**Figure 3:**
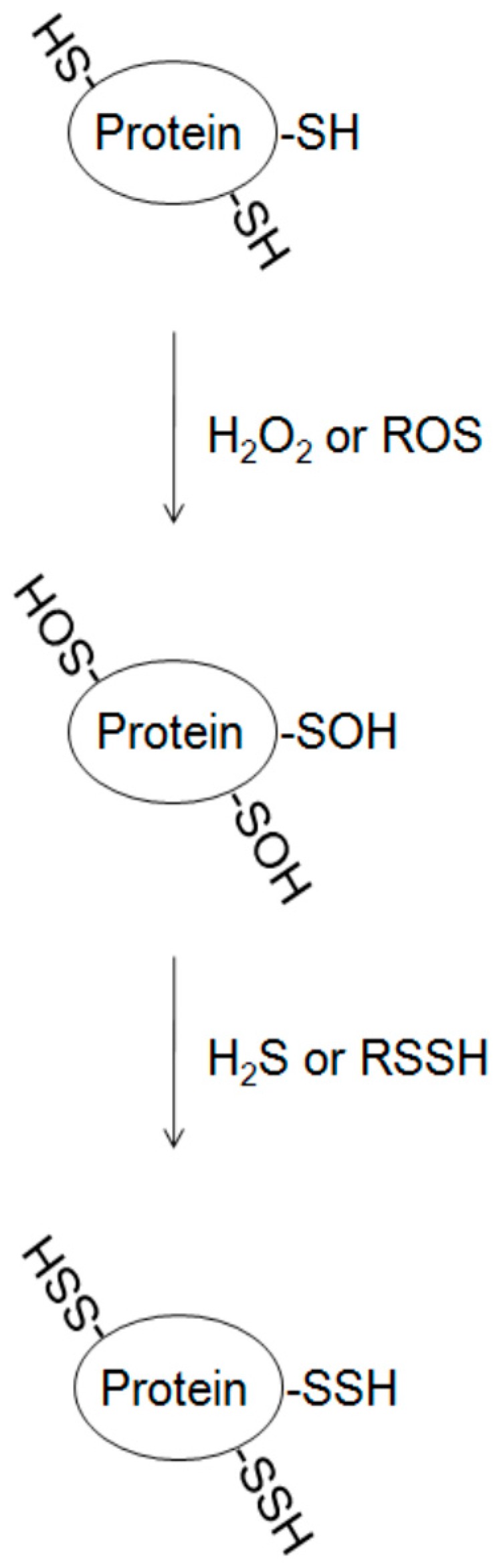
Potential mechanism of protein *S*-sulfhydration from oxidized cysteine in proteins. H_2_O_2_, hydrogen peroxide; H_2_S, hydrogen sulfide; ROS, reactive oxygen species; RSSH, hydropersulfides.

The direct reaction of cysteine and H_2_S is questioned. Thiols can be easily oxidized, and the presence of reactive oxygen species (ROS) inside the cells can react with the free thiols forming sulfenic (SOH) and sulfinic (SO_2_H) acids. It is predicted that H_2_S may first interact with oxidized cysteine in target protein resulting in the reduction of –SOH (*S*-sulfenylation) to –SSH (*S*-sulfhydration) [39] (Figure 3). *S*-sulfenylation, sulfenic acid modifications on cysteine residues in proteins, is reversible [58]. The propensity of cysteine residues to undergo oxidation is influenced mainly by three general factors: thiol nucleophilicity, the surrounding protein microenvironment, and proximity of the target thiol to ROS source [59]. Much evidence has demonstrated that the interaction of target protein with different ROS sources alters thiol status and induces spatial oxidation of cysteine residues [59,60]. The presence of H_2_O_2_ in the medium strengthened *S*-sulfhydration, further supporting this hypothesis [22,28]. Thus, cysteine *S*-sulfhydration can protect particular proteins from nucleophilic attack. Recent evidence also demonstrated that H_2_S-induced persulfide formation could not be the consequence of its reaction with protein disulfides, because incubation of the cells with diamide, an inducer of disulfide bond formation, attenuates H_2_S-induced *S*-sulfhydration [28]. Furthermore, the presence of different levels of ROS may alter the regulatory role of sulfane sulfur, such as thiosulfate, persulfides, thiosulfonate, polysulfides, polythionates, and elemental sulfur, on protein *S*-sulfhydration [61]. Further study of the regulatory mechanism will be helpful for understanding the susceptibility of protein thiol modification by *S*-sulfhydration.

### 2.5. Interaction of H_2_S and NO on Protein Modification

Both NO and H_2_S are important gasotransmitters and regulate diverse physiological functions through interaction [62,63,64]. H_2_S influences NO production and its metabolites by affecting NO synthase, and NO is also shown to alter H_2_S bioavailability by acting on H_2_S-generating enzymes. The same cysteine residue(s) in the target protein can be either *S*-nitrosylated or *S*-sulfhydrated [18]. More directly, H_2_S has been identified to intertwine with NO or its metabolites, forming various new compounds, such as thionitrous acid (HSNO), sulfinyl nitrite (HSNO_2_), or nitrosopersulfide (SSNO−) [64,65,66], depending on the concentration of H_2_S/NO and reaction conditions. The bioactivity of either NO or H_2_S is governed by concomitant formation of polysulfides and anionic S/N-hybrid species, which would subsequently attack protein for further modification [38].

## 3. The Donor Molecules for Protein *S*-Nitrosylation and *S*-Sulfhydration 

**Figure 4:**
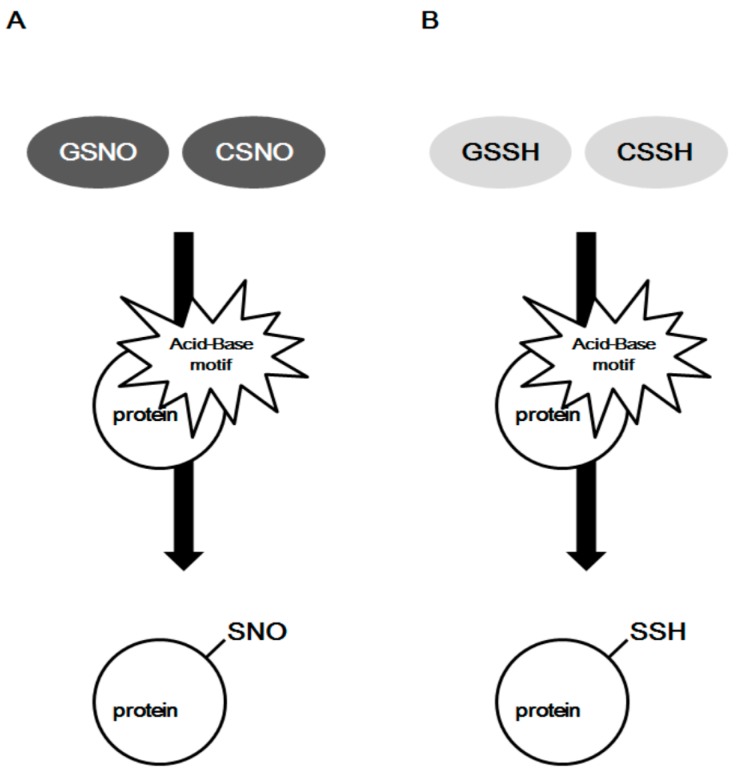
The forming mechanisms of protein S-nitrosylation and S-sulfhydration by physiological relevant donors. CSNO, nitrosocystiene; CSSH, cysteine persulfide; GSNO, nitrosoglutathione; GSSH, glutathione persulfide.

**Figure 5:**
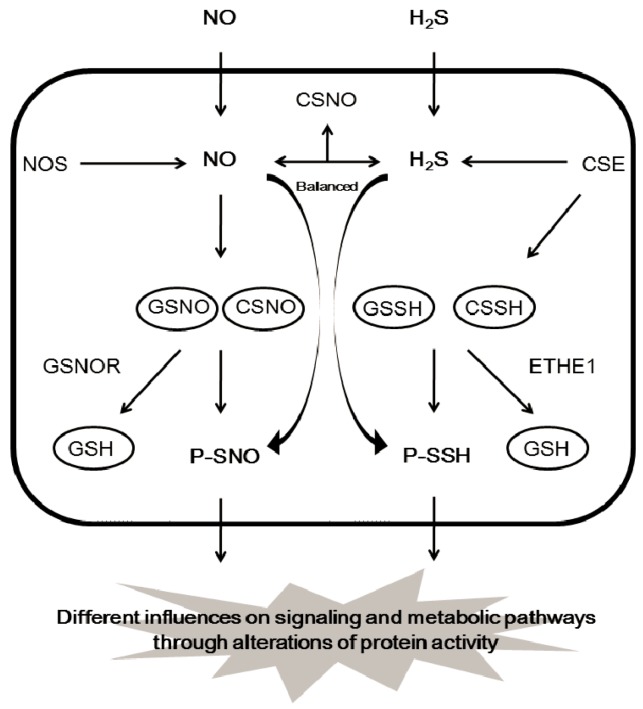
The pool of donors for protein S-nitrosylation and *S*-sulfhydration. ETHE1, mitochondrial persulfidedioxygenase; GSNOR, GSNO reductase.

The direct reaction of NO with thiols forming *S*-nitrosylation is unlikely, and the formation of an SNO is actually aided with higher oxides of NO-containing molecules, such as dinitrogen trioxide (N_2_O_3_), *S*-nitrosothiols, CSNO, and/or GSNO [67,68,69,70]. GSNO and CSNO are often seen to induce protein *S*-nitrosylation, and both can transfer their NO moiety to protein cysteine residues via transnitrosylation [33,34] (Figure 4A). It is not clear whether protein *S*-sulfhydration is an enzyme-catalyzed reaction or an automatic redox reaction. Nevertheless, many intermediates must be involved in the formation of protein *S*-sulfhydration. Persulfide RSSH including GSSH and cysteine persulfide (CSSH) would be the highly potential intermediate for the forming of protein *S*-sulfhydration due to their high electrophilic features [25,28,39] (Figure 4B). The concentration of GSSH and CSSH inside the cells is reported to be in the high micro molar range, which is positively correlated with H_2_S level in different tissues, such as brain, kidney, and liver. Under certain conditions, persulfides can release H_2_S following reduction by another species, including another persulfide, indicating that persulfides may facilitate sulfide storage and transport [71,72,73]. H_2_S may act as a marker for persulfides and polysulfides [61]. Endogenous GSSH is regulated by H_2_S-producing enzymes (CSE) or cystathionine beta synthase (CBS), since overexpression of CSE or CBS induces GSSH level and stimulates the formation of protein *S*-sulfhydration [39,74]. In addition, persulfide formation by CSE- and CBS-mediated CysSSCys metabolism is very active and can act as a source of biological persulfides [28]. In contrast, ETHE1 is reported to metabolize GSSH to GSH with simultaneous oxygen consumption [30]. It is not surprising that the level of *S*-sulfhydrated proteins, such as GAPDH, pyruvate carboxylase, and eNOS, depends on the expression and activity of H_2_S-generating enzymes within the cells [10,18,43]. The tissue or cell-specific protein *S*-sulfhydration may also exist due to the different level of enzymic activity of H_2_S-generating proteins. It can be predicted that, by mediating the generation of CSSH or GSSH, the persulfide trafficking among different proteins via transsulfhydration can occur (Figure 5). 

## 4. Prospection

Given the importance of protein *S*-sulfhydration in diverse cellular functions and pathophysiological responses, the regulatory mechanism of protein *S*-sulfhydration needs to be clarified. The interaction or competition between cysteine *S*-sulfhydration and *S*-nitrosylation in the same protein needs to be determined. Due to the instability and transition, development and improvement in protein *S*-sulfhydration detection technology and methodology is urgent for a better understanding of its formation and wide biological implications. By targeting protein *S*-sulfhydration, new drugs or solutions can be quickly developed for preventing and treating a wide range of diseases.

## Figures and Tables

**Table 1:** The proteins for transnitrosylation through protein-protein interaction.

Regulatory Protein	Target Protein	Reference
Trx1	Alpha enolase	[51]
	Heat shock cognate 71 kDa protein	[51]
	Peroxiredoxin-1	[51]
Glyceraldehyde-3-Phosphate Dehydrogenase (GAPDH)	Deacetylating enzyme sirtuin-1	[54]
	Histone deacetylase-2	[54]
	DNA-activated protein kinase	[54]
	B23/nucleophosmin	[56]
Haemoglobin	Anion exchanger AE1	[55]

## Supplementary Materials

Supplementary File 1

## Abbreviations

3D, 3-dimensional; 3MST, 3-mercaptopyruvate sulfurtransferase; ASK1, apoptosis signal-regulating kinase 1; BSA, biotin switch assay; CBS, cystathionine beta-synthase; CSE, cystathionine gamma-lyase; CSNO, nitrosocystiene; CSSH, cysteine persulfide; DNA-PK, DNA activated protein kinase; DTT, dithiothreitol; EDRF, endothelial-deriving relaxing factor; ETHE1, mitochondrial persulfidedioxygenase; GSNO, nitrosoglutathione; GSNOR, GSNO reductase; GSSH, glutathione persulfide; H_2_O_2_, hydrogen peroxide; H_2_S, hydrogen sulfide; HDAC2, histone deacetylase 2; KO, knockout; MMTS, methyl methanethiosulfonate; MSBT, methylsulfonylbenzothiazole; NF-kB, nuclear factor kappa b; N_2_O_3_, dinitrogen trioxide; NO, nitric oxide; NO^+^, nitrosoniumcation; PTM, post translational modification; ROS, reactive oxygen species; RSNO, nitrosothiol; RSSH, hydropersulfides; SIRT1, deacetylating enzyme sirtuin 1; SNO, *S*-nitrosothiol; SNOSID, SNO site identification; SOH, sulfenic; SO_2_H, sulfinic; Trx1, thioredoxin 1; WT, wild-type.
