# Supplementary material for: H2S-Mediated Protein S-Sulfhydration: A Prediction for Its Formation and Regulation
Source: Molecules. 2017 Aug 11;22(8):1334. doi: 10.3390/molecules22081334 (PMC6152389; doi:10.3390/molecules22081334)
Supplement: Supplementary File 1 [file molecules-22-01334-s001.zip › molecules-22-01334-g005.pdf]

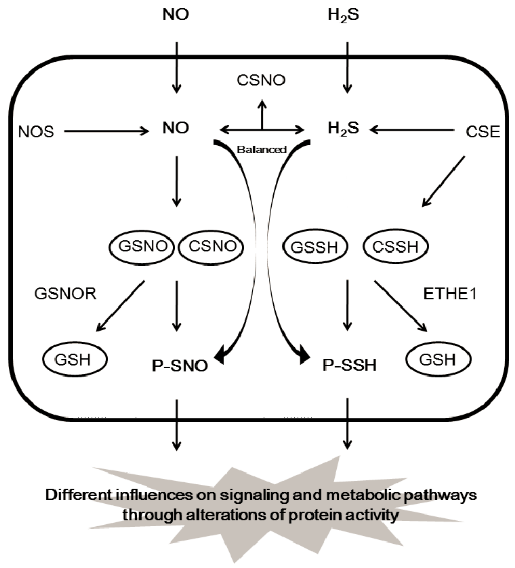

NO
H₂S
CSNO
NOS
NO
H₂S
CSE
Balanced
GSNO
CSNO
GSSH
CSSH
GSNOR
ETHE1
GSH
P-SNO
P-SSH
GSH
Different influences on signaling and metabolic pathways
through alterations of protein activity
