# Supplementary figures and images for: H2S-Mediated Protein S-Sulfhydration: A Prediction for Its Formation and Regulation
Source: Molecules. 2017 Aug 11;22(8):1334. doi: 10.3390/molecules22081334 (PMC6152389; doi:10.3390/molecules22081334)

**A**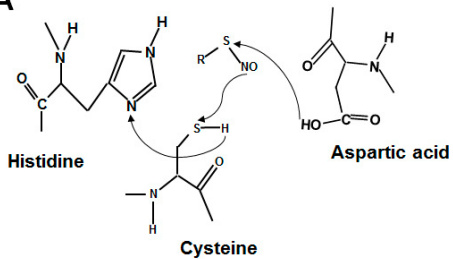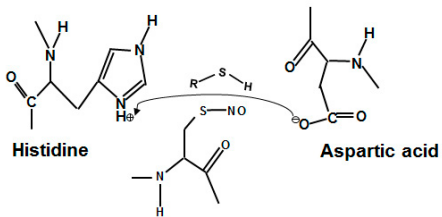**B**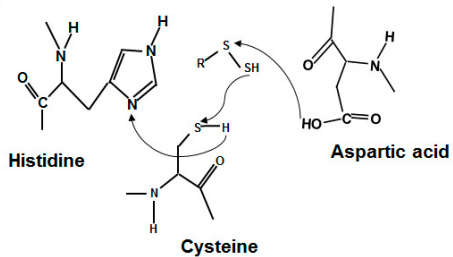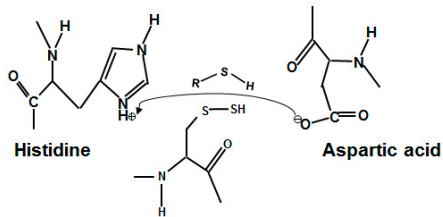

Supplement: Supplementary File 1 [file molecules-22-01334-s001.zip › molecules-22-01334-g001.pdf]

**A**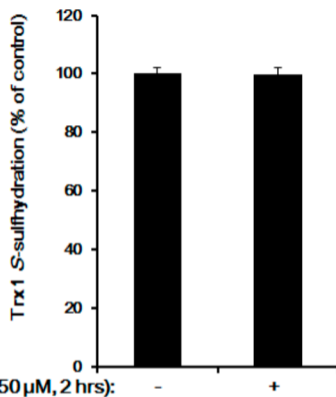

S-sulphydrated  
Trx1

Load  
Trx1

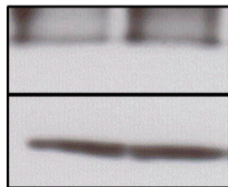**B**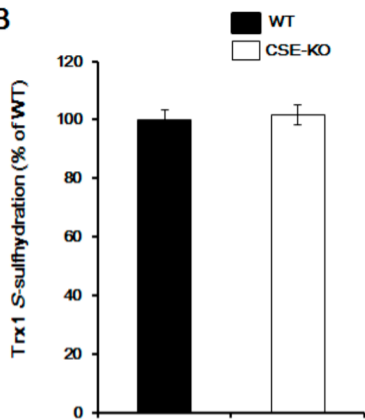

S-sulphydrated  
Trx1

Load  
Trx1

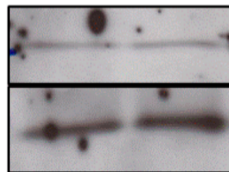

Supplement: Supplementary File 1 [file molecules-22-01334-s001.zip › molecules-22-01334-g002.pdf]

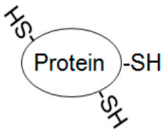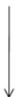

$\text{H}_2\text{O}_2$  or ROS

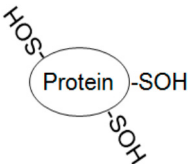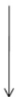

$\text{H}_2\text{S}$  or RSSH

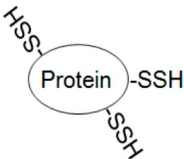

Supplement: Supplementary File 1 [file molecules-22-01334-s001.zip › molecules-22-01334-g003.pdf]

A

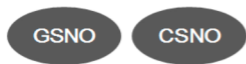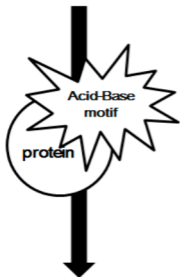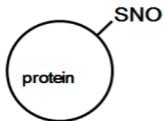

B

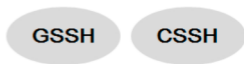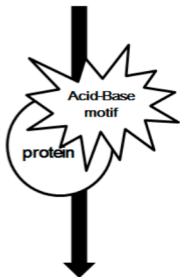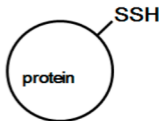

Supplement: Supplementary File 1 [file molecules-22-01334-s001.zip › molecules-22-01334-g004.pdf]
